# High-throughput process development from gene cloning to protein production

**DOI:** 10.1186/s12934-023-02184-1

**Published:** 2023-09-15

**Authors:** Manman Sun, Alex Xiong Gao, Xiuxia Liu, Yankun Yang, Rodrigo Ledesma-Amaro, Zhonghu Bai

**Affiliations:** 1 National Engineering Research Center of Cereal Fermentation and Food Biomanufacturing, Jiangnan University, Wuxi, 214112 China; 2 Department of Bioengineering and Imperial College Centre for Synthetic Biology, Imperial College London, London, SW7 2AZ UK; 3 Division of Life Science, The Hong Kong University of Science and Technology, Hong Kong, China; 4 Key Laboratory of Industrial Biotechnology, School of Biotechnology, Ministry of Education, Jiangnan University, Wuxi, 214122 China; 5 Jiangsu Provincial Research Center for Bioactive Product Processing Technology, Jiangnan University, Wuxi, 214122 China

**Keywords:** Recombinant protein, Process development, High-throughput technology, High-throughput culture

## Abstract

In the post-genomic era, the demand for faster and more efficient protein production has increased, both in public laboratories and industry. In addition, with the expansion of protein sequences in databases, the range of possible enzymes of interest for a given application is also increasing. Faced with peer competition, budgetary, and time constraints, companies and laboratories must find ways to develop a robust manufacturing process for recombinant protein production. In this review, we explore high-throughput technologies for recombinant protein expression and present a holistic high-throughput process development strategy that spans from genes to proteins. We discuss the challenges that come with this task, the limitations of previous studies, and future research directions.

## Background

Recombinant proteins are utilized across a wide range of industries including food, chemistry, biopharmaceuticals, and biomaterials [1]. According to the latest protein expression market research report, the global protein expression market is rapidly growing, especially after the COVID-19 outbreak. The market size was valued at USD 3.18 billion in 2022 and is expected to expand at a compound annual growth rate (CAGR) of 9.36% from 2023 to 2030 [2]. Traditionally, process development for recombinant protein production follows standard procedures established many decades ago. This process includes the selection of appropriate genetic components from existing expression toolkits through trial and error [3, 4], followed by optimizing process-related parameters one by one in shake flasks, microtiter plates (MTPs) or laboratory-scale bioreactors [5–8]. Undoubtedly, this is a time-consuming process, and it usually takes several years for a protein to develop from laboratory research to industrial production. However, the need for rapid protein production in the post-genomic era has led to the reshaping and optimization of previous process development strategies. To keep up with the demand, laboratories and enterprises are integrating high-throughput technologies into their workflows. This integration not only accelerates the production of recombinant proteins but also streamlines the entire process from gene to protein, creating a more comprehensive and time-efficient process.

Over the past decades, numerous methods have been developed to enable the high-throughput construction of strains [9–12]. The integration of high-throughput protein detection methods and cultivation platforms, such as MTPs, microbioreactors, and parallel fermentation systems, has made the cultivation, screening, and optimization process more efficient [13–16]. Furthermore, the adoption of novel process optimization strategies has significantly reduced the process development timeline. Herein, we review the adaptations and developments made by academic and industrial laboratories to accelerate protein production. A holistic high-throughput development strategy from genes to proteins is proposed and recommended to ensure robust and cost-effective development of the protein production processes.

### High-throughput construction of strain libraries

At present, various expression systems including bacterial, yeast, insect, and mammalian cells have been developed for recombinant protein production [3, 12]. Since the target protein can have different origins and characteristics, it is challenging to predict the best expression system and still involves a significant degree of trial and error [17, 18]. Furthermore, even after selecting the expression system, choosing expression elements, such as promoters and signal peptides, is still a challenge. To maximize protein yields, it is recommended to create a library (large starting population with genetic diversity). To date, various methods have been developed for the high-throughput construction of expression strain libraries, including random mutagenesis, laboratory evolution, artificial synthesis, knockouts, and overexpression [19–22]. Among them, constructing a clone library through the combination of expression elements is the most commonly used method, which enables the systematic optimization of elements for protein expression [15, 23].

**Fig. 1:**
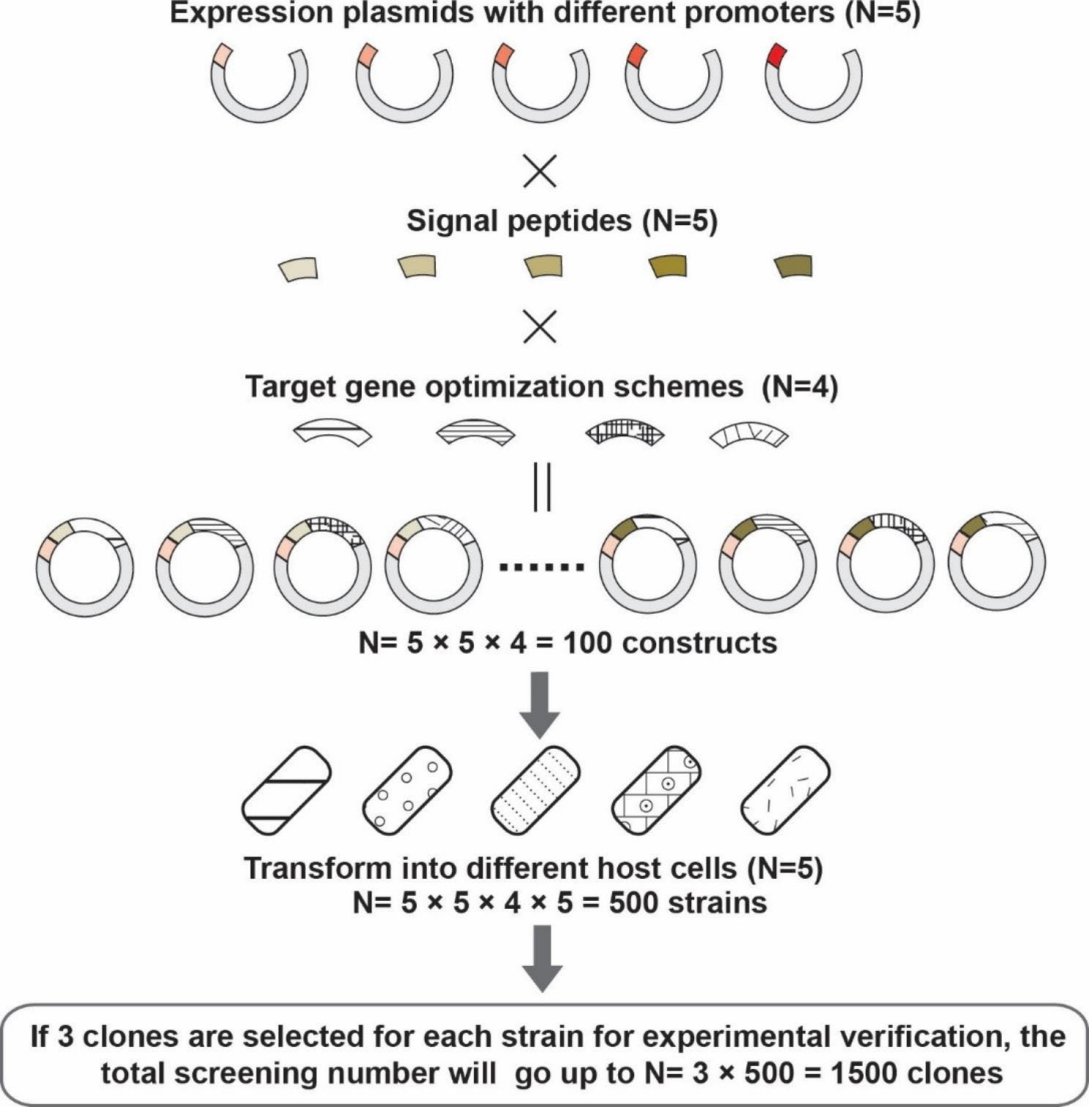
Construction of recombinant strain library. A massive amount of recombinant strains is constructed according to the trial and error principle for achieving the most efficient expression combination. In this example, five promoters, five signal peptides, four target gene optimization schemes, and five phenotypes of host strain are considered for choosing the optimal clone candidate

Accordingly, a given protein sequence can generate a large number of clones through the combination of promoters, signal peptides, target gene sequences, and host cells, which can be as high as n ≥ 1000, to establish the best candidate strain [15] (Fig. 1). Over the years, various DNA assembly techniques have been developed to simplify and reduce the cost of constructing expression vectors [24]. Based on the basic principle, these methods are classified into the following three main categories: Restriction enzyme-based cloning, recombination-based cloning, and ligation-independent cloning.

### Restriction enzyme-based cloning

**Fig. 2:**
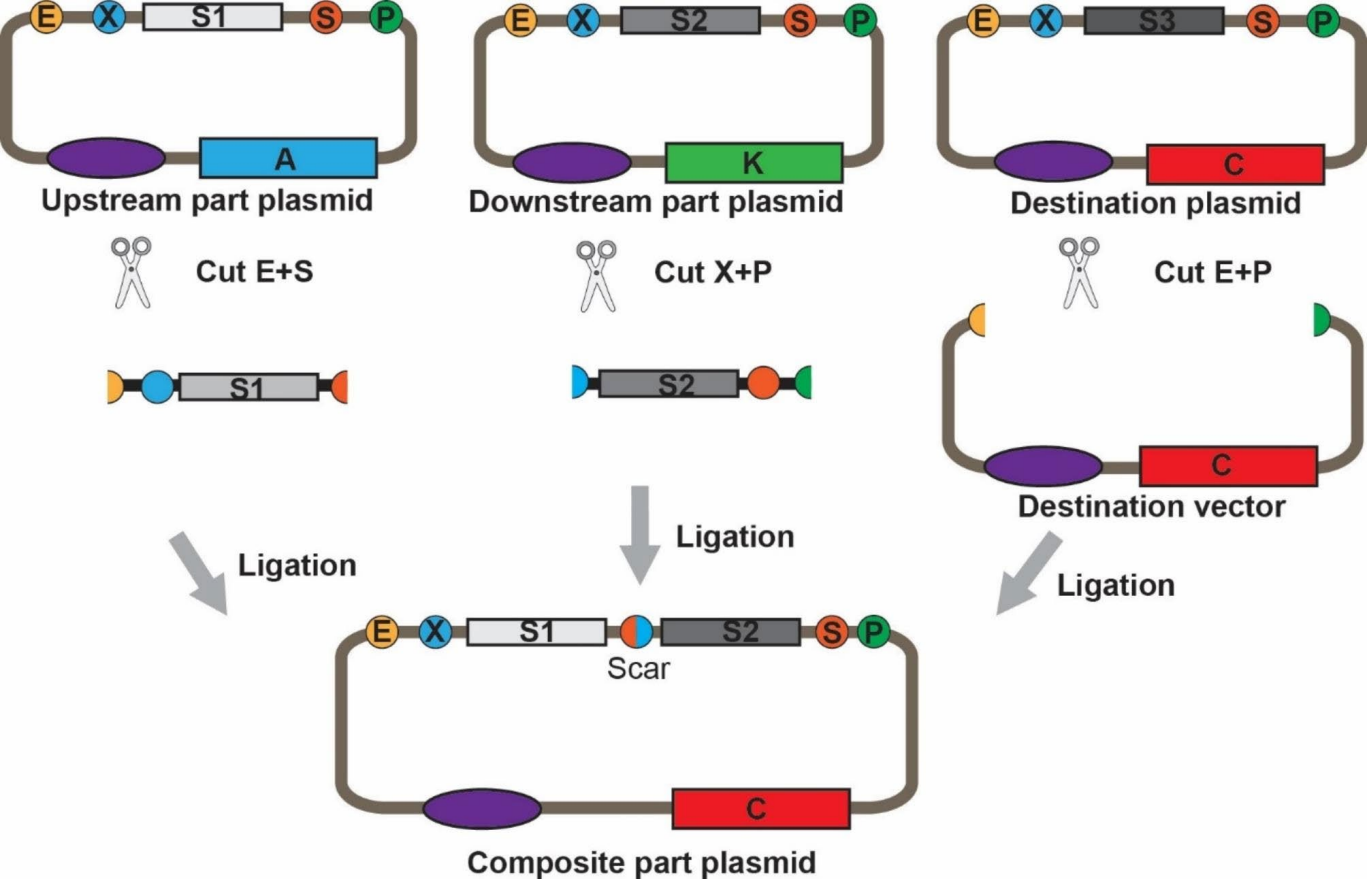
Schematic diagram of the 3 A assembly process for assembling BioBrick parts. To perform 3 A assembly, the destination plasmid, the upstream part plasmid and the downstream part plasmid must have different antibiotic resistance markers from each other. Abbreviations are as follows: A = ampicillin resistance gene, K = kanamycin resistance gene, C = chloramphenicol resistance gene, S1, 2, 3 = sequence 1, 2, 3, E = *Eco*RI, X = *Xba*I, S = *Spe*I, P = *Pst*I. 3 A assembly works as follows: Digest the upstream part plasmid with *Eco*RI and *Spe*I. Digest the downstream part plasmid with *Xba*I and *Pst*I. Digest the destination vector with the *Eco*RI and *Pst*I. Then, all digested plasmids are mixed, ligated, and transformed to a solid plate supplemented with antibiotic corresponding to the destination vector resistance marker for selection. The scar represents the mixed *Xba*I/ SpeI site

Restriction enzyme-based cloning is a classic and widely-used method for molecular cloning that involves the digestion of DNA by restriction enzymes (RE) and the subsequent ligation of the resulting fragments. Several common systems such as the Flexi Cloning system and Golden Gate are all based on this principle [24]. This method gained renewed attention in 2003 when Knight, T. proposed the BioBrick standard for the physical assembly of biological parts [25]. The BioBrick standard requires two special sequences for each BioBrick part, called the prefix and suffix sequences, containing REs for *Eco*RI/*Xba*I and *Spe*I/*Pst*I, respectively. Among these REs, *Xba*I and the *Spe*I are isocaudomers, allowing the assembly of composite BioBrick parts [26, 27]. The key innovation of BioBrick assembly is that any two BioBrick parts can be assembled, and the resulting composite itself is also a BioBrick part that can be assembled again. Later, in 2011, Shetty et al. further developed this method and proposed the three antibiotic assembly (3 A assembly) method for the construction of BioBrick parts [27]. The 3 A assembly requires three plasmids for molecular cloning and the destination plasmid must carry a different antibiotic-resistance gene from the other two plasmids. The schematic diagram of the 3 A assembly was shown in Fig. 2. Finally, positive clones can be easily obtained through antibiotic resistance-based positive and negative selection. Compared with previous RE-based cloning, the 3 A assembly eliminates the time- and labor-intensive steps such as column cleanup and agarose gel purification during plasmid construction, increasing the throughput of molecular cloning [27–29]. This system also supports the iterative assembly of genetic components, making it an ideal tool for high-throughput construction of expression element combinations for recombinant protein production [26, 27, 30]. However, the use of 3 A assembly introduces two additional amino acids, which limits its use in scenarios with strict requirements on the protein sequence.

### Recombination-based cloning

**Fig. 3:**
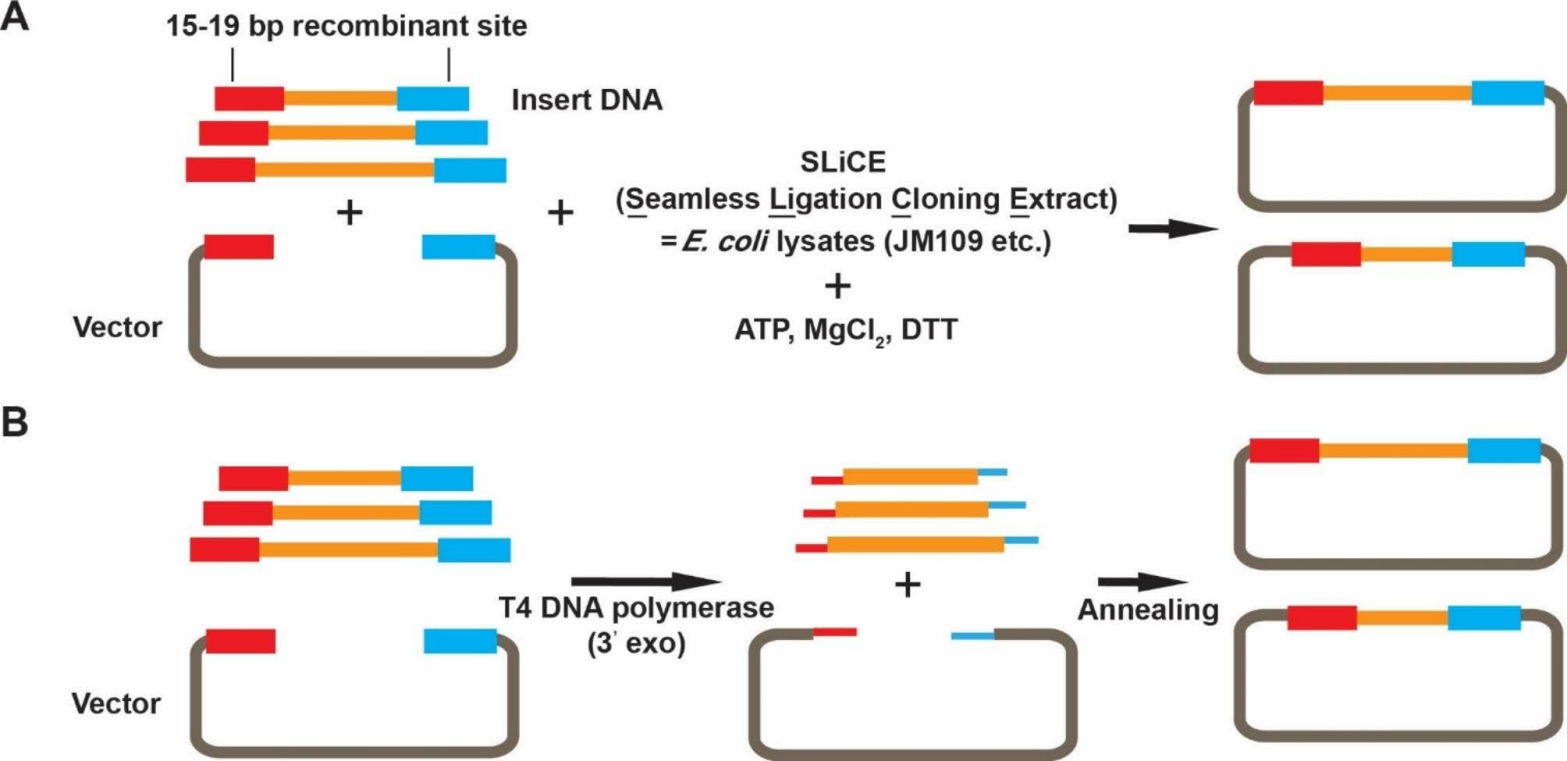
Schematic diagram and the principle of the SLiCE method and LIC method. (**A**) Overview of SLiCE cloning. Target genes are flanked by 15–19 bp recombination sites. Laboratory *E. coli* strains´ SLiCE-mediated recombination between the homologous arms generates the final vector. (**B**) A schematic for the production of recombinant DNA through LIC cloning. The linearized expression vector and target gene containing complementary tails are digested by T4 DNA polymerase (3’ exo) and then transformed into *E. coli* for in vivo ligation after annealing

The development of recombinant cloning systems has revolutionized the construction of multiple plasmids. Among them, Gateway is probably the most successful and widely used [12]. This technology exploits a site-specific recombination system originally observed in lambda phage to transfer heterologous DNA sequences between two vectors with flanking compatible recombination attachment (*att*) sites [31]. To further improve the throughput and specificity of Gateway cloning, adjustments have been made, such as changing the sequence or length of att sites to clone multiple genes or fragments simultaneously [12]. However, high costs limit the widespread use of this method. To address this issue, Zhang et al. developed an alternative recombinant cloning system called SLiCE (Seamless Ligation Cloning Extract), which directly utilizes the homologous recombination activity in cell lysate prepared from the *Escherichia coli* DH10B strain expressing a lambda prophage Red/ET recombination system, enabling the assembly of multiple DNA fragments into vectors in a single in vitro reaction [32]. This method was further improved by Motohashi by directly utilizing cell lysates from several common laboratory *RecA*^*−*^*E. coli* strains, including DH5α, JM109, DH10B, XL10-gold, and Mach1 T1 [33] (Fig. 3A). Since the SliCE method does not require the use of REs and ligases, and many standard laboratory bacterial strains can serve as the source of SliCE extracts, SliCE has become a simple, efficient, and ultra-low-cost alternative to commercial kits for performing high-throughput cloning [34].

### Ligation-independent cloning

Ligation-independent cloning (LIC) is a method developed 30 years ago that enables directional cloning of any fragment after generating a DNA sequence containing a single-strand (SS) complementary end [35]. The LIC method mainly uses the exonuclease activity of T4 DNA polymerase or T5 exonuclease to generate SS complementary tails [36–38]. Here, we give a schematic for the production of recombinant DNA using T4 DNA polymerase-based LIC (Fig. 3B). As LIC does not require REs, ligases, or recombinases, it has become an inexpensive and easily adaptable method for high-throughput cloning. Hitherto, many commercially available kits based on the LIC principle have been developed, including In-Fusion from Clontech and Gibson Assembly from NEB [9]. Later, to further improve the versatility and efficiency of LIC, some improved methods have been developed, such as sequence and ligation–independent cloning (SLIC) [39], improved SLIC [40], Nicking Endonucleases based LIC (NC-LIC) [41], and uracil-excision based cloning [42, 43]. Recently, the coupling of LIC with automatic-control devices and micro-well plates has also further improved the efficiency of LIC-based plasmid construction [44, 45].

### High-throughput analytical technology for protein expression

High-throughput protein expression analysis is critical in the early stages of process development. However, for many recombinant proteins, accurate quantification of their expression levels still relies on labor-intensive SDS-PAGE analysis, especially for those without specific detection methods. While several high-throughput protein analysis platforms, such as Octet™ (Pall ForteBio Corp, USA), LabChip GXII (Perkin-Elmer Inc.), and the E-PAGE™, have been developed [15], they are still in the early stage of adoption and not widely used. For most laboratories, the common method for high-throughput protein analysis is the fusion of a protein of interest with a fluorescent protein. Due to its small molecular weight and high fluorescence intensity, *Aequorea victoria* green fluorescent protein (GFP) and its mutants, such as enhanced green fluorescent protein (EGFP) and superfolder GFP (sfGFP), are widely used as fusion markers [46–48]. As shown in the work of Kovacevic and co-workers, they correlated the activity of glucose oxidase (GOx) with GFP fluorescence [47]. In recent years, split-EGFP technology has also been developed. Instead of full-length GFP, split-EGFP technology divides the GFP into several fragments, allowing for the target protein to be fused with a small GFP fragment, thereby minimizing interference on the activity of the target protein [49]. This system has been successfully used for the high-throughput detection of a thermostable esterase Aaeo1 expression library (25,000 clones) in *E. coli* [50]. Another alternative strategy is the bicistronic design (BCD)-based transcriptional fusion with fluorescent proteins, where the translation of the target gene is coupled with a response gene encoding a fluorescent protein [51]. This system allows for the detection of target protein expression by monitoring fluorescence intensity, without introducing additional amino acids into the target protein [52]. Furthermore, the use of fluorescence-activating and absorption-shifting tags (FAST) emerges as another novel alternative for high-throughput protein analysis. FAST tags can be attached to target proteins, allowing for rapid, specific, and highly sensitive detection, thereby enhancing the efficiency and precision of high-throughput protein expression analysis [53].

Although the fusion expression strategy is convenient and accurate, it still has limitations in detecting transiently expressed or fast-degrading proteins with short half-lives [54]. To overcome this issue, biosensors such as the STEP sensor (sensor for transiently expressed proteins) have been developed, providing a solution for the high-throughput detection of protein expression, especially for transiently expressed proteins [55].

### High-throughput cultivation platform

**Fig. 4:**
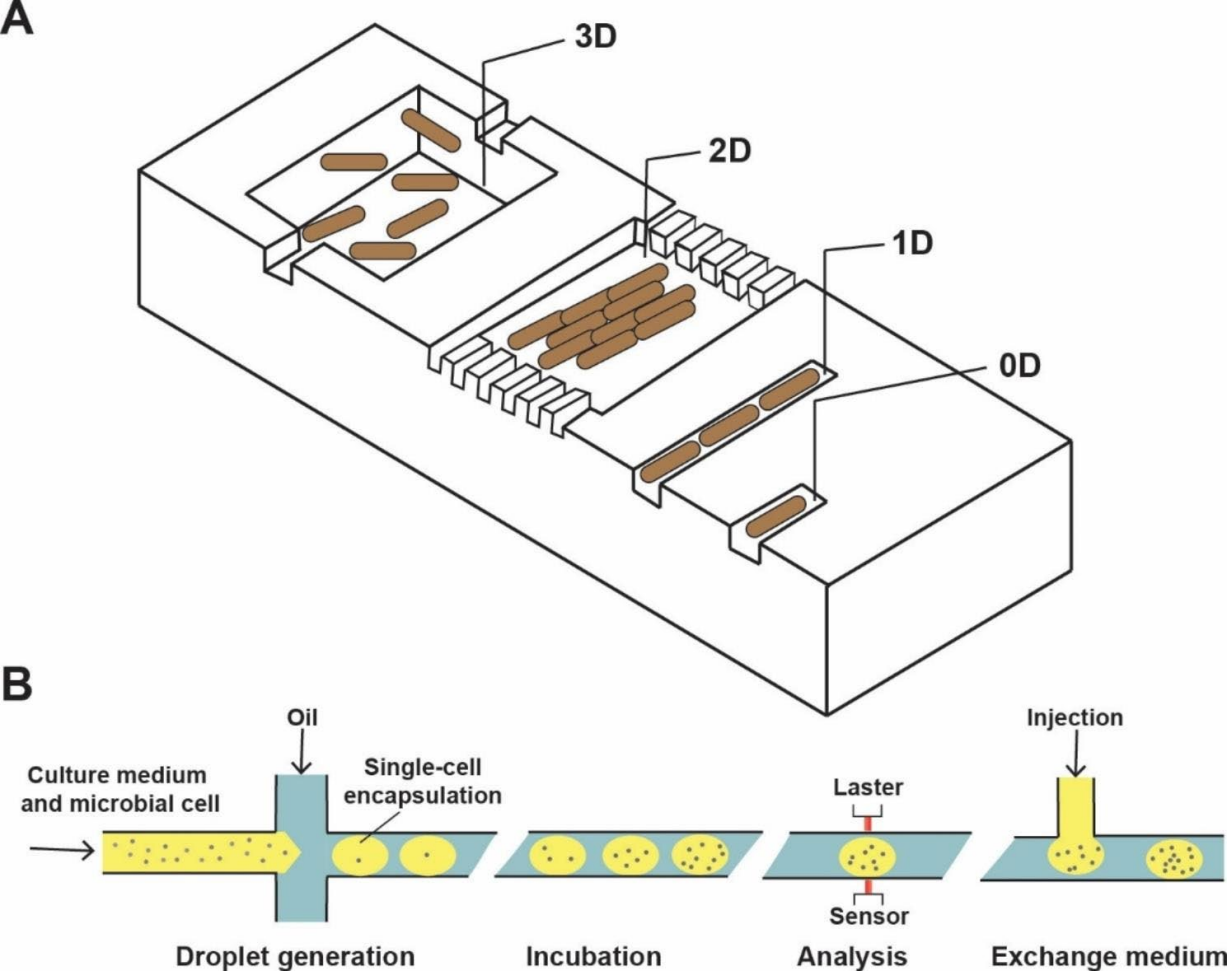
Microfluidic-based micro-cultivation system. (**A**) Overview of the geometric principles of microbial single-cell reactors. Nanoliter chambers for 3D cultivation, picoliter 2D chambers to hold cell monolayers, and femtoliter channels for 1D linear single-cell rows and single-cell traps. (**B**) Droplet-based microfluidic micro-cultivation system

The development of a reliable and cost-effective high-throughput cultivation platform is crucial due to its time-consuming and costly nature. Over the past decades, various miniaturized culture devices have been developed, enabling cultivation at milliliter, microliter, or even picoliter scales [14, 56]. A good example of these devices is the microfluidic-based cultivation system [56]. To date, various microfluidic bioreactors, single-cell habitats, trapping cavities, and cultivation chambers have been developed. Based on the cells′ degree of freedom, these microfluidic culture devices can be classified into different dimensions (ranging from 0-dimensional (0D) to 3D) [14, 56] (Fig. 4A). Despite their advantages, microfluidic-based cultivation systems increase the risk of contamination due to the use of continuous single-phase flow [57]. To overcome this problem, researchers further developed microdroplet technology [58, 59] (Fig. 4B). By separating the carrier fluid from the culture medium and encapsulating microbial cells in droplets, microdroplet technology eliminates contamination [59]. These innovative microdroplet systems have been successfully used for microorganism enrichment [60], high-throughput characterization and screening of strains [19, 61], adaptive evolution [59], etc. Another representative cultivation platform is the microliter-level microtiter plates (MTPs) system, the advantages of high-throughput, easy-to-operate, and low-cost advantages of MTPs make it a widely used cultivation platform [15, 62, 63]. To date, various MTPs formats (6 − 1,536 wells) have been developed and many auxiliary devices such as pipetting robots, autosamplers, and microplate readers have been made compatible with MTPs [63]. MTPs have now become a cheap alternative to shake flasks for strain cultivation.

To better meet the needs of high-throughput process development of protein production, a vast number of miniaturized bioreactors, such as the miniature 10-ml stirred-tank bioreactor, 10-mL scale microbioreactor, 5-ml Applikon microreactor, 3-ml Biocurve, and µ-bioreactor system BioLector have also been developed [62], and a few of these have been successfully commercialized. In Table 1, we listed and compared several commercial mini-bioreactor systems. Depending on the culture broth mixing mechanism, they can be categorized as bubble column- or microplate-based mini-bioreactors and stirred mini-tank bioreactors [15]. These platforms facilitate strain screening: They not only allow high-throughput screening of strains under controlled conditions, but also some of them can collect a wealth of process information online (protein titer, protein quality, specific productivity, cell fitness, or robustness) for each clone. However, these miniaturized bioreactors were considered not ideal for high-throughput optimization due to the lack of independent feeding systems and the inability to achieve high cell densities. Recent advances in some mini-reactors such as the microbial Ambr15 fermentation system (Ambr15f) have overcome this limitation. The pumped liquid line in Ambr15f can feed each vessel as needed, making it a good scale-down model for fermentation parameter optimization [64].

In addition to the above miniaturized bioreactors, researchers have also developed a variety of parallel fermentation systems, such as the microbial Ambr250 system [65], the 4×1 L Biocuber system [23], Multifors 2 system [66], BioXplorer system [67], multi-bioreactor system BIOSTAT® Qplus, and Dasgip parallel bioreactor system [68, 69] (Table 2). These systems consist of multiple small stirred bioreactors that operated simultaneously, they offer several advantages over traditional single bioreactor setups, including increased throughput and reduced resource consumption, and reduced space requirements [62]. With larger culture volumes (usually 50-1000 mL) than micro-bioreactors (< 15 mL), parallel fermentation systems enable us to perform a complete downstream analysis. These systems have now been widely used in fermentation parameter optimization [15, 23].

**Table 1:** Comparison of several commercial mini-bioreactor systems and their applications in modern bioprocess development/research

Name	BioLector	Micro-24	Micro-matrix	2mag bioreactor 48	Ambr15f
**Characteristics**	Microplate-based bioreactor; Operates with non-invasive optical sensors; Real time culture monitoring; Can be upgraded to fully automated units	Bubble column-based bioreactor; 24 simultaneous cultures with independent control of each reactor; individual pH, DO, and temperature sensors ; Real time monitoring	Microplate-based mini-bioreactor; 24 simultaneous cultures with independent control of each reactor; Individual liquid addition; Real time monitoring	Microplate-based mini-bioreactor; 48 parallelized mini reactors; Non-invasive real-time measurement of pH and DO	Micro-stirred tank bioreactor; Single-use pH and DO sensors; Available with temperature compensation for low-temperature application; Real time monitoring
**Working volume**	0.8–1.5 mL/well	3–7 mL/column	1–7 mL/well	8–15 mL/well	10–15 mL/vessel
**Availability of feed**	Require an external liquid handling station	Integrated liquid feed individually per column	Integrated liquid feed individually per bioreactor	Require the integration of a pipetting robot	Integrated liquid feed individually per vessel
**Application areas**	Cell and strain screening; Growth characterization analysis; Medium screening and optimization; Culture condition optimization; Systematic biology; Gene/proteomics studies	Clone screening; Optimizing temperature, pH, and DO conditions for cell growth; Optimizing the induction condition for protein expression; Optimizing medium composition; Process development studies	Screening cell-line, microbial, and/or yeast libraries; Process development studies; Process optimization studies; Small volume cultivations	Clone selection; Media screening and optimization; Optimization of the process design; Studies of gene and protein expression	Clone selection; Media and feed optimization; Process intensification; Development of advanced cell therapies
**Reference**	10.1186/1472-6750-11-22 10.1186/1475-2859-8-31	10.1002/btpr.522 10.1002/bit.22031	10.2144/btn-2019-0063	10.1007/s00449-022-02798-6	10.1002/btpr.2534
**Website of suppliers**	https://www.m2p-labs.com/	http://www.pall.com/main/biopharmaceuticals	http://www.applikon-bio.com	https://www.2mag.de/en/produkte-e/bioreactor-e/bioreactor-48-e.html	http://www.tapbiosystems.com

**Table 2:** Summary of basic characteristics of several commercial parallel fermentation systems

Name	Ambr250 system	Biocuber system	Multifors 2
**Characteristics**	Contain 12 or 24 fully featured single-use mini bioreactors; Individual control for each bioreactor vessel; Integrated liquid feed individually per bioreactor; Real time culture monitoring	Contain 4 independently controlled bioreactors; Integrated liquid feed individually per bioreactor; Real time culture monitoring	Contain 6 independently controlled bioreactors; Integrated liquid feed individually per bioreactor; Real time culture monitoring
**Working volume**	100–250 mL	300 mL to 1 L	150 mL to 1 L
**Application areas**	Applicable for process development; Process optimization; Scale-down studies; Cell culture and microbial fermentation	Applicable for the process development of bacteria, yeast, mammalian cell cultures; Clone screening; DoE optimization	Applicable for the process development of microorganisms and mammalian cell cultures; Clone screening; DoE optimization
**Reference**	10.1002/biot.201700766	10.1007/s00253-022-11918-x	10.3389/fbioe.2021.695306
**Website of suppliers**	https://www.sartorius-stedim-tap.com/tap/cell_culture/ambr_250.htm	http://www.bioyd.com/	https://www.infors-ht.com/en/bioreactors/bench-top-bioreactors/multifors2/
**Name**	**BIOSTAT® Qplus multi-bioreactor system**	**BioXplorer 100/400**	**Dasgip parallel bioreactor system**
**Characteristics**	Fully independent control of up to 12 expandable culture vessels; Specifically designed for early process development and multivariable process optimization; Offers a broad range of measurement and automation features	Parallel processing of 4 or 8 independent reactors; Integrated individual liquid and gas feeds with options for gas mixing; Real time culture monitoring	Fully independent control of up to 16 expandable culture vessels; All control systems can be accessed remotely by one or more remote operators simultaneously
**Working volume**	150 mL to 1 L	20 to 100 mL; 20 ml to 400 ml	1.3 to 4.3 L
**Application areas**	Applicable for microbial, mammalian, insect and plant cell growth studies; Clone screening; DoE optimization; Small-scale expression of recombinant proteins	Microbial fermentation; C1 Gas fermentation and cell culture; Process development, DoE optimization	Applicable for process development of bacteria, yeast, fungi, and mammalian cell cultures; DoE optimization
**Reference**	10.1038/nmeth.f.340	10.3389/fbioe.2021.695306	10.1186/1472-6750-12-96
**Website of suppliers**	https://www.sartorius.com/en	https://helgroup.com/products/bioreactors/	http://www.dasgip.com

### High-throughput strategy for process optimization

#### Optimization of the medium composition

After a production strain has been chosen, it becomes necessary to further optimize the components of the cultivation medium. Traditional one-factor-at-a-time (OFAT) methods in shake flasks are low-throughput and fail to consider interactions between medium components. To mitigate this limitation, a combination of high-throughput cultivation platforms such as micro-bioreactors or MTPs with experimental design (DoE) can be utilized [23, 70–72]. DoE enables the study of interactions between variables and reliable prediction of results in unexplored conditions [73, 74], which can significantly reduce the number of necessary experiments and increase experimental throughput. Moreover, model predictive control (MPC) can also be integrated into this kind of DoE-based framework, allowing the precise, real-time adjustment of cultivation conditions based on predictive models. This comprehensive approach takes into account the interdependencies between different medium components and process parameters, thus facilitating the efficient determination of the optimal medium composition [75, 76].

#### Optimization of the fermentation parameters

Fermentation parameters such as dissolved oxygen (DO), feeding rate, pH, and agitation speed significantly affect protein expression. Considering the importance of these parameters at the industrial scale, it is essential to optimize them in stirred bioreactors [77]. Traditional optimization methods based on iterative experiments in laboratory-scale bioreactors are expensive and time-consuming. Therefore, stirred mini-tank bioreactors or parallel fermentation systems combined with the DoE strategy are increasingly used. For instance, Janakiraman et al. optimized monoclonal antibody production in CHO cells by using the Ambr system and DoE to determine the optimal growth temperature, production temperature, and pH [78]. Our previous study optimized the fermentation parameters for OmlA antigen production by combining the Biocuber system and DoE [23]. Recent advancements in high-throughput stirred bioreactors, such as the Ambr 15f system software’s compatibility with DoE packages and the BioPAT® MFCS/win module’s facilitation of automated optimization experiments, have greatly simplified the DoE-based process optimization, enhancing reliability and reproducibility [64, 79]. Successful applications include the optimization of malaria vaccine production in *Pichia pastoris* [79].

Building on these innovations, MPC can further optimize the process by enabling dynamic, real-time adjustments of fermentation parameters [75, 76]. Simultaneously, a comprehensive digital infrastructure enhances data management, analytics, and automation, leading to a more efficient and reliable DoE-based fermentation process optimization [80].

#### Current limitations

Although high-throughput process development has shown satisfactory results, there are still some limitations that need to be addressed. Firstly, there is a lack of systematic integration of high-throughput technology to cover the entire process of protein production. Previous applications of high-throughput technology have mainly focused on a small part of the overall protein production process [81]. Although several researchers have begun to use high-throughput technology in an integrated manner, the practice still needs to be more widespread to cover the whole process [23, 82, 83]. Secondly, most previous studies have paid little attention to the reliable scale-up of the fermentation process. They either finish after establishing the fermentation process in miniaturized bioreactors or just copy the fermentation parameters settings established in these scale-down bioreactors to large-scale ones [84, 85]. Microbial phenotypic heterogeneity may be aggravated during the scale-up process due to the formation of gradients such as oxygen and substrates in large-volume vessels, which have also been overlooked and not thoroughly discussed [86]. Finally, process development strategies need to be adjusted according to the existing high-throughput platforms. For instance, most production strains in previous studies were screened solely based on protein titers in uncontrolled cultivation platforms [19, 20]. With the development of micro-bioreactors with online monitoring systems, multi rounds of screening with multiple performance criteria evaluations (protein titer, protein quality, specific productivity, cell fitness, or robustness) should be adopted. Additionally, as the throughput of the culture platform increases, the combination of experimental variables and experimental design should be carefully explored to obtain the best results [87].

#### High throughput process development for recombinant protein production

**Fig. 5:**
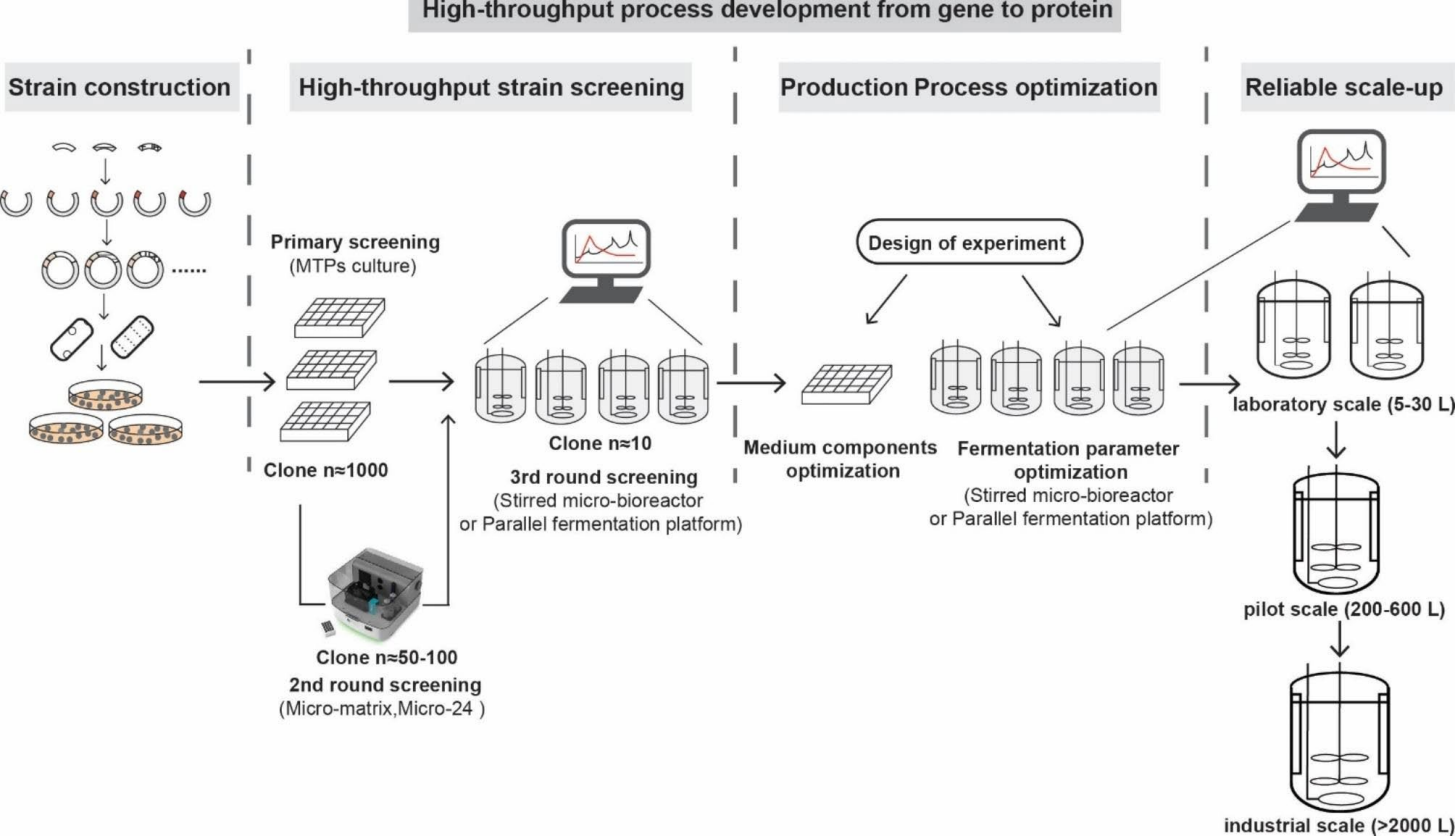
Representative flow chart of high-throughput process development from gene cloning to protein production in the current bio-industry sector. Model predictive control (MPC) and a comprehensive digital infrastructure can be integrated to accelerate this process

Here, we summarized a representative development process for recombinant protein production. The holistic process includes high-throughput clone construction and screening, high-throughput production process optimization, and reliable scale-up of the production process (Fig. 5).

#### High-throughput screening

Constructing expression libraries is widely utilized to identify the optimal expression element combination for the production of a given protein. As described above, various high-throughput cloning methods have been developed, allowing us to construct large libraries of strains in a short time. For the screening of the production strain from a large pool of candidates (n ≈ 1000), a multi-round of screening strategy is highly beneficial [13, 15, 88, 89] (Fig. 5). The first round of screening is typically performed in an uncontrolled MTPs system to select the most promising clones based on selection criteria such as protein titer (g/L), which would reduce the number of clones for the following round (n ≈ 50–100). At this stage, the glucose limited fed-batch technology, a strategic maneuver that optimizes the fermentation process by precisely controlling the supply of glucose, can be adopted, to fully unleash the production potential of clones [90]. The second round of screening can be conducted in a highly parallel controlled platform, such as micro-24 or micro-matrix, further reducing the number of candidate clones (n ≤ 10). Finally, the performance of these selected strains is comprehensively evaluated in cultivation systems closer to actual production, such as stirred micro-bioreactor and parallel fermentation system, to identify the best production strain. It is worth noting that the strain ranking in the parallel fermentation system and micro-bioreactor may differ from that in the MTPs, reflecting the impact of environmental factors on microbial performance [23, 89].

#### High-throughput production process optimization

Once the production strain has been selected, further experimental optimization is needed to determine the optimal production medium and fermentation parameters for the production of the target protein. Considering a large number of experimental variables, a high-throughput cultivation platform combined with the DoE strategy, is strongly recommended at this stage [71, 91] (Fig. 5). In this context, the integration of MPC and a comprehensive digital infrastructure can further enhance the optimization process. Following this strategy, the experimental design optimization of medium components is suggested to be performed in MTPs, while the optimization of fermentation parameters is preferably performed in stirred micro-bioreactors or parallel fermentation systems [23]. These combined high-throughput optimization strategies, supported by MPC and a comprehensive digital infrastructure, allow us to quickly identify the critical process variable and determine their “design space” [15, 23, 92]. Ultimately, the optimal setting level for the key variable can be easily determined through a limited number of experiments.

#### Reliable scale-up of the production process

The fermentation process established in the scale-down model needs to be scaled up to a larger scale bioreactor for further evaluation or actual commercial production (Fig. 5). To ensure the reliability of the scale-up, it is important to adopt a suitable scale-up criterion. Constant oxygen mass transfer coefficient (k_L_a), constant specific power input (P/V), constant impeller tip speed, and constant dissolved oxygen concentration are four commonly used scale-up criteria in the fermentation industry [93]. However, due to the complexity of the cell culture process and the varied characteristics of recombinant proteins, it seems that no criterion can be universally applied with a high success rate [94]. The actual selection of scale-up criteria should be based on the specific characteristics of the fermentation process. Generally, a constant k_L_a strategy is recommended for scaling up aerobic microorganisms [23]. Whereas constant P/V is often used as a scale-up criterion for early industrial penicillin fermentation and low-energy input fermentation [95], and this strategy is limited in fermentation processes that require high-energy input, such as the recombinant *E. coli* culture [96]. Constant tip speed is ideal for scaling up antibiotic fermentation and evaluating the possibility of hyphal rupture in the fermentation of branched yeast, filamentous bacteria, and fungi [97], but it is less useful for single-cell fermentation. When heat transfer is a limiting factor for fermentation scale-up, such as high-density fermentation of *Pichia pastoris* using methanol as a carbon source, scale-up based on constant dissolved oxygen concentration is preferred [98]. Additionally, strain and inducer modifications, cell physiology manipulations, and bolus feeding with pulses strategy can also be adopted to reduce cell phenotypic heterogeneity during the scale-up process [86]. Finally, it is worth noting that, the experimenter’s intuition and expertise are also crucial in the scale-up process [99].

## Conclusion and future outlook

In this article, we reviewed the high-throughput technologies that have been developed and applied to the recombinant expression of proteins. We also proposed a holistic high-throughput process development strategy. To further accelerate the process development for protein production, there is still much work to be done. A primary imperative is the broad-scale integration of automated laboratory processes. This integration, achieved by harmonizing automated sample preparation with cultivation platforms and aligning them with high-throughput analytical tools, serves to reduce human error while enhancing laboratory efficiency [100, 101]. Connecting these disparate elements of the protein production workflow allows us to create a unified, efficient, and predominantly automated process that could redefine the standards of future protein production. Potential areas for future developments lie in refining detection tools and experimental equipment. For instance, biosensors, a staple in metabolic engineering, could be further optimized for protein expression detection, especially in the case of secreted proteins. The introduction of disposable, pre-sterilized bioreactors could mitigate sample contamination and decrease labor-intensive preparation. In addition, as the application of various high-throughput technologies increases, the generation of data multiplies and solid systems to manage, store and analyze the obtained results need to be developed. A comprehensive digital infrastructure can be established for managing, sharing, and analyzing experimental data throughout the development process [80]. Moreover, the creation of a public database of protein expression data and the introduction of bioinformatics analysis are also necessary. The public database can collect and share the conditions and results of protein expression from laboratories worldwide. By inferring rules based on bioinformatics analysis of previous data, researchers can predict which expression elements and culture conditions may be successfully used for protein expression with specific characteristics, thus greatly reducing the workload of clone construction and process optimization [102]. Finally, it is expected that new artificial intelligence (AI) and machine learning (ML) techniques will play a critical role in such development. They can improve process efficiency, enhance product quality, and reduce production costs. Additionally, AI and ML can support scale-up, data integration and visualization, and automation, leading to faster and more cost-effective production of bio-products [103, 104].

## Data Availability

Not applicable.
